# The determinants and consequences of adult nursing staff turnover: a systematic review of systematic reviews

**DOI:** 10.1186/s12913-017-2707-0

**Published:** 2017-12-15

**Authors:** Mary Halter, Olga Boiko, Ferruccio Pelone, Carole Beighton, Ruth Harris, Julia Gale, Stephen Gourlay, Vari Drennan

**Affiliations:** 1 Faculty of Health, Social Care and Education, Kingston University and St George’s, University of London, Cranmer Terrace, London, SW17 0RE UK; 2 Florence Nightingale Faculty of Nursing and Midwifery, King’s College London, James Clerk Maxwell Building, 57 Waterloo Road, London, SE1 8WA UK; 3 Faculty of Health, Social Care and Education, Kingston University and St George’s, University of London, Kingston Hill, Surrey, KT2 7LB UK; 4 Department of Management, Faculty of Business & Law, Kingston University, Kingston Hill, Surrey, KT2 7LB UK; 5 National Guideline Alliance, Royal College of Obstetricians and Gynaecologists, 27 Sussex Place, Regent’s Park, London, NW1 4RG UK

**Keywords:** Nursing staff, Nurses, Personnel turnover, Workforce, Review, systematic, Research design (data quality, data reporting), Determinants, Consequences

## Abstract

**Background:**

Nurses leaving their jobs and the profession are an issue of international concern, with supply-demand gaps for nurses reported to be widening. There is a large body of existing literature, much of which is already in review form. In order to advance the usefulness of the literature for nurse and human resource managers, we undertook an overview (review of systematic reviews). The aim of the overview was to identify high quality evidence of the determinants and consequences of turnover in adult nursing.

**Methods:**

Reviews were identified which were published between 1990 and January 2015 in English using electronic databases (the Cochrane Database of Systematic Reviews, MEDLINE, EMBASE, Applied Social Sciences Index and Abstracts, CINAHL plus and SCOPUS) and forward searching. All stages of the review were conducted in parallel by two reviewers. Reviews were quality appraised using the Assessment of Multiple Systematic Reviews and their findings narratively synthesised.

**Results:**

Nine reviews were included. We found that the current evidence is incomplete and has a number of important limitations. However, a body of moderate quality review evidence does exist giving a picture of multiple determinants of turnover in adult nursing, with - at the individual level - nurse stress and dissatisfaction being important factors and -at the organisational level - managerial style and supervisory support factors holding most weight. The consequences of turnover are only described in economic terms, but are considered significant.

**Conclusions:**

In making a quality assessment of the review as well as considering the quality of the included primary studies and specificity in the outcomes they measure, the overview found that the evidence is not as definitive as previously presented from individual reviews. Further research is required, of rigorous research design, whether quantitative or qualitative, particularly against the outcome of actual turnover as opposed to intention to leave.

**Trial registration:**

PROSPERO Registration 17 March 2015: CRD42015017613.

**Electronic supplementary material:**

The online version of this article (10.1186/s12913-017-2707-0) contains supplementary material, which is available to authorized users.

## Background

Nurses leaving their jobs or leaving the profession, known more commonly in human resource terms as turnover [1], is an issue of concern in all health care systems [2]. Low retention rates of health care professionals, including qualified nurses, are detrimental to the delivery of health care systems and population health [3]. In high income countries retention of nurses and other health care professionals is also viewed as an important health human resource strategyto reduce demand for and therefore migration of nurses from health care systems in low income countries [3]. Data from the North America have been used to suggest that many high income countries are experiencing or predicting growth in demand for qualified nurses over the next decade [4, 5]. In those high income countries facing shortage of supply of experienced qualified nurses such as England, reducing turnover and improving retention rates has become an important workforce development strategy [6].

Definitions of nurse turnover differ in operational practice and in research studies [7]. Turnover can be described as voluntary (including retirement) or involuntary, [8] avoidable or not avoidable; [1] and can be internal, that is leaving for another nursing or non-nursing job in the same organisation or external, that is leaving for another nursing or non-nursing job in a different organisation [9]. It can also refer to nurses leaving the nursing profession but remaining on a nurses’ register, or leaving a nurses’ register, [10] or to a number of combinations of the above descriptors [1]. It is in this context of a lack of consistency in the definition and measurement of turnover that the rate of nurse turnover has been estimated at between four and 54% intending to leave internationally [11]. In a review of studies which used the same method of measuring turnover and its costs (the Nursing Turnover Cost Calculation Methodology [12–14]), the rates reported in primary studies still varied from 15% in Australia, 20% in Canada, 27% in the USA to 44% in New Zealand [7].

In England, in addition to the usual nurse turnover rates, a significant increase in demand for nurses qualified to work with general adult patients has occurred in recent years [15]. This has been attributed to the fall in commissioned nurse education places, [16] to high profile reports highlighting serious quality and safety issues [17, 18] and to the publication of evidence-based guidelines on safe nurse staffing levels [19]. Nurses working in general adult health services, in comparison to those working in paediatric or psychiatric services, are the largest group of nurses in all countries [4, 20, 21]. It should be noted that there is diversity between countries in whether the education for nurse registration or licensure is generic to all populations or specialist to particular groups such as children [22]. In this paper nurses working in general adult health services are described as those in ‘adult nursing’ for brevity.

The human resources literature offers us a large number of antecedents of actual turnover found on meta-analysis, including those in the groupings of personal characteristics, satisfaction, work experience, external environment factors, behavioural predictors and cognitions and behaviours about the withdrawal process [23]. Such antecedents are variously represented in a number of well-developed models of turnover, including those describing organisational contexts and psychological (behavioural) explanations of turnover where characteristics lead to intentions leading to turnover, [24] as well as those indicating the importance of the ‘webs of relationships in which employees are situated’, for example the role of centrality in social networks as a moderator to the psychological processes ([25], p1177) or the impact of dispositional traits such as locus of control and proactive personality, particularly in explaining wide variance in the intentions – actual turnover relationship. Specific to nursing, turnover is recognised to be “complex and multifaceted with factors affecting every sector of health care” [26] and several conceptual models have been put forward, recognising the decades of work on nurse turnover [27]. These models variously recognise a plethora of reasons why nurses leave or state their intention to leave, [27], although they have been broadly described in three categories: motivational characteristics, social characteristics and characteristics of the work context, although the latter has been less well explored in the research [25]. In these models, nurse turnover is also reported to have consequences, mainly reported as negative in terms of cost, compromise to patient safety and effect on remaining staff [2]. As these consequences take us full circle to antecedents, we have included these in this paper.

Our awareness of the existence of models within and outside of the nursing literature, and the large literature their authors call upon, led us to undertake a preliminary stage of review - making an assessment of potentially relevant literature specific to nursing and its size for review [28] - when we were commissioned to carry out a review of the adult nurse turnover literature. Using Medline alone at this stage we identified a large body of reviews relevant to the study’s objectives that indicated that nurse and human resource managers would be faced by a plethora of reviews [29, 30], many of which were not conducted according to reviews guidance [26]. Against this background, we conducted an overview which is a systematic review of systematic reviews [31].

This paper reports on this overview, which aimed to identify high quality evidence of the determinants and consequences of turnover in nurses working in the field of adult health care services and bring that evidence together into one place to highlight where strong enough evidence to support managerial decisions exists and where gaps in the evidence may indicate the need for further research, particularly when considered in the context of the broader management literature regarding turnover.

## Methods

We based the review methods on the Preferred Reporting Items for Systematic review and Meta-analysis Protocols (PRISMA-P) 2015 statement [32] and Cochrane Handbook for Systematic Reviews of Interventions [31, 33].

### Criteria for considering studies for review

This overview included data from qualitative, quantitative and mixed methods reviews published in English from 1990 onwards. Inclusion criteria were as follows:

- Population: the reviews should be focused on those delivering adult nursing (i.e. licensed or registered) in health care services (both in hospital and community health services) in developed economies (according to the definition of the International Monetary Fund [34]).
- Issue of interest: the reviews should have examined the determinants and/or consequences of turnover in nurses working in adult health services.
- Comparison: any comparators, if any, used within the included reviews.
- Outcomes: the reviews should report measures of determinants and/or consequences of adult nursing turnover outcomes. The outcomes included in the review depended on the types of outcomes examined in the retrieved reviews, but were anticipated to include turnover / retention rate and intention to leave/stay.
- Review design I (for all stages of the overview): any form of literature review (e.g. either systematic or non-systematic reviews) which had been peer-reviewed, contained a statement of review, reported its search strategy and/or inclusion/exclusion criteria, reported either empirical findings or a list of included primary studies and included a methodological quality assessment of its included primary studies.
- Review design II (for narrative synthesis): any review that had carried out and reported a methodological quality assessment of its included primary studies.

Exclusion criteria were as follows: Reports from any types of primary studies; reviews published in language other than English; reviews that did not evaluate adult nursing turnover as described in the inclusion criteria or presented data on nurses working across settings that could include the care of children or in specific mental health settings; reviews that did not report empirical findings; reviews published only in abstract form; any form of literature review using informal and subjective methods to collect and interpret evidence, commentaries and non peer-reviewed reviews; any review in which majority of included articles were non-peer reviewed publications and reviews that did not report an appraisal of the quality of the studies they included.

### Search methods for identification of studies

We searched the Cochrane Database of Systematic Reviews, MEDLINE (Ovid), EMBASE (Ovid), Applied Social Sciences Index and Abstracts –ASSIA, CINAHL plus (EBSCO) and SCOPUS –V.4 (Elsevier) from 1990 to 2015 (searches conducted January 2015). Search strategies were guided by a systematic approach to the research questions [35] and a Medline search strategy was developed (Table 1) and converted or modified to run on other databases (Additional file 1). We identified additional studies by searching on PubMed by using the “related citations” algorithm and screening the reference lists of included studies for other reviews [36].

**Table 1:** Medline search strategy and number of articles found −17/01/2015

Search line number	Search concept	Search terms	Number of retrieved articles
1	Nursing	exp/Nursing staff	34,054
2		exp Nursing Care/	58,012
3		exp Nurses/	41,985
4		(nurse or nurses or nursing).tw.	175,720
5		1 or 2 or 3 or 4	229,449
6	Turnover	exp Personnel Turnover/	2969
7		(turnover or (leave adj5 (nurse or nurses or nursing)) or (leaving adj5 (nurse or nurses or nursing)) or (retention adj5 (nurse or nurses or nursing)) or (retain adj5 (nurse or nurses or nursing)) or (stay adj5 (nurse or nurses or nursing))).tw.	44,114
8		6 or 7	45,826
9	Systematic reviews	meta-analysis.pt.,ti,ab,sh.	63,056
10		(meta anal$ or metaanal$).ti,ab,sh.	76,516
11		((methodol$ or systematic$ or quantitativ$) adj5 (review$ or overview$ or survey$)).ti,ab,sh.	66,923
12		(medline or embase or index medicus).ti,ab.	57,130
13		((pool$ or combined or combining) adj (data or trials or studies or results)).ti,ab.	10,736
14		literature.ti,ab.	350,875
15		9 or 10 or 11 or 12 or 13 or 14	457,235
16		15 and review.pt.,sh.	217,379
*17*	*Reviews of Nursing and Turnover*	*5 and 9 and 16*	*173*
*18*		limit 217 to english language	*170*

### Selection of studies

The results of the electronic search were downloaded into an Excel spreadsheet. After duplicate articles were removed, relevant reviews were selected according to eligibility criteria using a two-step screening process:

- Title and abstract screening. Two authors (FP and MH) reviewed in parallel the titles and abstracts of all the articles resulted to ascertain their eligibility for full text retrieval. Disagreements were resolved by peer discussion and a third view from the project lead (VMD) if required.
- Full-text screening. Two reviewers (MH and OB or OB and CB) read in parallel all the selected full-text articles citations to analyse whether they meet all the inclusion/exclusion criteria. Any discrepancies between the two reviewers will be resolved in discussion with the third reviewer (FP where MH and OB had read in parallel and MH where OB and CB had read in parallel).

### Data extraction

Three authors (MH, OB and CB) extracted data from the included reviews using a predefined extraction form and spreadsheet on: *general characteristics of the review*: e.g. author(s), year, geographical scope, research area, and authors’ aims/ research question(s); *descriptive characteristics*: e.g. type of review (design); selection criteria to include primary studies, number and study designs of articles incorporated in the reviews, outcome measures; *results*: every determinant or consequence in the included reviews, listed by the outcome measured, the direction of findings against that outcome and the references for the primary studies; *main conclusions,* using the review authors’ words, and *limitations*, as noted by the review authors. Discrepancies were resolved through discussion among the data extractors.

### Assessment of methodological quality

The 11-point Assessment of Multiple Systematic Reviews (AMSTAR) checklist [37] was used to assess the quality of each included review. This tool has been widely used in previous similar overviews and it is considered to be a valid and reliable instrument [38]. Using the AMSTAR scale two authors appraised each included paper. Reviews that scored eight or higher were considered at low risk of bias (high quality), between five and seven were at moderate risk of bias (moderate quality) and four or less were at high risk of bias (poor quality).

The primary studies included in each review were also listed and compared across the reviews to assess the degree of overlap in the reviews comprising our overview.

### Data analysis

Because of the heterogeneous nature of the focus, inclusion criteria and outcome measures of the included studies data were analysed thematically. Following the detailed reading involved for data extraction, the resultant spreadsheet was examined and a thematic index of determinants and consequences developed (using reviews that met our inclusion criteria for including a methodological assessment of their primary studies as well as those that did not). The thematic index (Additional file 2) was applied to each data extraction and four main groupings of determinants (individual, professional, interpersonal and organisational) and one of cost consequences was used to analyse across reviews, using Microsoft Excel 2010 to record the decisions applied for all reviews considered (Additional file 3). A narrative account of the findings from the reviews containing an assessment of the methodological quality of included primary studies has been structured using the risk of bias in the review as the primary grouping level and the thematic content analysis as the second level, also drawing on the number and quality of the included primary studies. In this way we aim to describe the findings by ‘weight of evidence’ [39]. The systematic review protocol was registered with PROSPERO (International database of prospectively registered systematic reviews in health and social care) PROSPERO 2015: CRD42015017613 [40].

## Results

### Review selection, study characteristics and quality assessment

#### Review selection

**Fig. 1:**
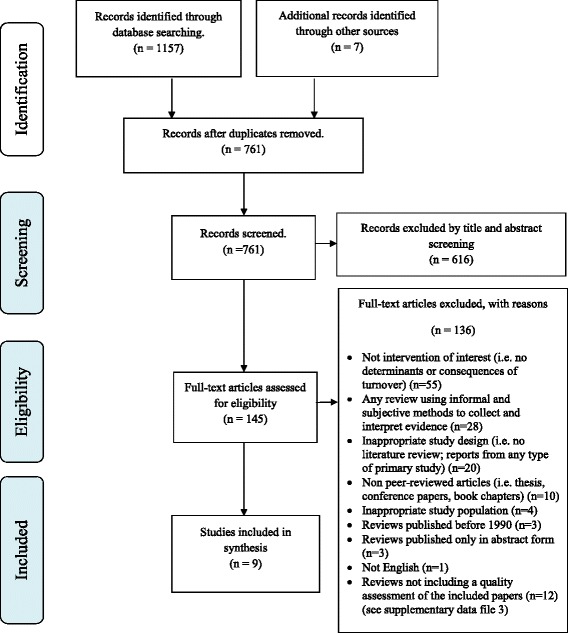
PRISMA Flow Diagram

The flow chart representing study selection, including reasons for exclusion, is summarised in Fig. 1. A total of nine reviews met the inclusion criteria and were included in the review.

Additional file 4 provides a list of citations for the excluded studies in the final stage of the selection process, as well as a table describing the characteristics of the 12 studies excluded only on the basis of not having presented a methodological assessment of the quality of the included articles.

#### Study characteristics

The characteristics of the nine included systematic reviews are presented in Table 2. The included reviews were all published in English; four were authored from the United States of America [41–44], and one each from Australia, [45] Canada, [46] Finland, [11] Singapore [47] and UK [48]. Of these, six had been published since 2010. Eight reviews had been published in four academic journals about nursing (Journal of Nursing Management, [41–43, 46] Journal of Advanced Nursing, [44] International Journal of Nursing Studies [48] and Nursing Ethics [45]), and one in the International Journal of Evidence-Based Healthcare. None was a Cochrane review.

**Table 2:** General characteristics of the included systematic reviews

Author(s) (year)	Aim(s) Research question(s)	Selection criteria used to include primary studies (PICOS)		Scope		Type, number, and quality of included studies as reported by the author(s)		Review authors’ summary of findings
				1. Geography				
				2. Time limit				
				3. Language				
Chan et al. (2013) [41]	To examine and describe the published empirical research on nurses’ intention to leave their current employment or the profession.	*P*	RNs working in non-specialty wards	1	International (by USA)	**Total number**	**31**	The reasons for nurses’ intention to leave are complex and influenced by many factors, categorised as individual and organizational factors. Individual factors are job satisfaction, burnout and demographic factors, whereas organizational factors comprise work environment, culture, commitment, work demands and social support. This review indicates that job satisfaction is the most influential.
		*I*	determinants (aspects, factors)	2	2001–2010	Quantitative	29	
		*C*	Not stated	3	English	*Experimental (quasi)*	*–*	
		*O*	Intention to leave			*Observational*	*29*	
		*S*	All types of peer-reviewed primary studies (no literature reviews, dissertations)			Qualitative	*–*	
						Mix-Methods	*–*	
						Other	2	
						*Quality*		
						No details available.		
						Critical Review of Quantitative		
						Research Worksheet (Miller 2006) [51]		
Coomber & Barriball (2007) [48]	To explore the impact of job satisfaction components on intent to leave and turnover for hospital-based nurses.	*P*	Hospital nurses	1	International (by UK)	**Total number**	**9**	From the four themes discussed, three were organisational factors (leadership, stress and pay) and only one an individual/ demographic factor (educational attainment).
		*I*	determinants	2	1997–2004	Quantitative	6	The empirical evidence shows that stress and issues concerning leadership consistently exert both direct and indirect effects on job satisfaction and intent to leave
		*C*	Not stated	3	Not stated	*Experimental (quasi)*	*–*	
		*O*	intent to leave/turnover			*Observational*	*6*	
		*S*	Primary and secondary research (no literature reviews)			Qualitative	3	
						Mix-Methods	*–*	
						Quality		
						No details available.		
Cowden et al. (2011) [46]	To examine the relationship between managers’ leadership practices and staff nurses’ intent to stay in or to leave their current position.	*P*	Staff nurses and their managers	1	International (by Canada)	**Total number**	**23**	The findings of the present study support a positive relationship between transformational leadership, supportive work environments and staff nurses intent to remain in their current position. Stated intentions to stay are strongly predictive of retention and turnover. Relational leadership styles attentive to the individual needs of the nurse promote staff nurses intentions to stay.
		*I*	determinants	2	1985–2010	Quantitative	22	
		*C*	Non stated	3	English	*Experimental (quasi)*	*–*	
		*O*	Intention to stay (behavioural intention)			*Observational*	*22*	
		*S*	Peer-reviewed qualitative or quantitative studies			Qualitative	–	
						Mix-Methods	1	
						Other	–	
						“..All studies were rated as moderate or strong” p.468		
						Tool adapted from several existing frameworks (Cummings and Estabrooks 2003 [52], Wong and Cummings 2007 [53], Lee and Cummings 2008 [54])		
D’Ambra & Andrews (2014) [42]	To determine the impact of incivility as experienced by new graduate nurse and negative effect of incivility on retention and patient care, and identify current organisational strategies suggested by that literature to mitigate the occurrence of incivility.	*P*	Newly graduated RNs	1	International (by USA)	**Total number**	**16**	The reasons for nurses’ intention to leave are complex and influenced by many factors, categorised as individual and organizational factors. Individual factors are job satisfaction, burnout and demographic factors, whereas organizational factors comprise work environment, culture, commitment, work demands and social support. This review indicates that job satisfaction is the most influential.
		*I*	Interventions to reduce workplace incivility	2	2002–2012	Quantitative	3	
		*C*	–	3	English	*Experimental (quasi)*	*–*	
		*O*	Recommended			*Observational*	*3*	
		*S*	All types of peer-reviewed primary studies (no literature reviews, dissertations)			Qualitative	2	
						Mix-Methods	–	
						Other	11^	
						^very unclear/no tabulation of papers		
						*Quality*		
						No details available.		
						Tool adapted from two existing frameworks (Schmidt and Brown 2012 [55]; Fineout-Overholt and Melnyk 2009 [56]).		
Flinkman et al. (2010) [11]	To review and critique the published empirical research on nurses’ intention to leave the profession	*P*	RNs or nurses with different educational background	1	International (by Finland)	**Total number**	**31**	A number of variables influencing nurses’ intention to leave the profession were identified, including demographic, work-related and individual-related variables. The proportion of nurses considering or intending to leave the profession varied considerably across studies.
		*I*	Determinants	2	1995-Jul 2009	Quantitative	31	The timeframe for leaving intention also varied
		*C*	Not stated	3	English, Swedish, Finnish	*Experimental (quasi)*	*–*	
		*O*	Intention to leave			*Observational*	*31*	
			Nurses’ retention			Qualitative	–	
		*S*	Not stated (no editorials, opinions or discussions)			Mix-Methods	–	
						Other	–	
						*Quality*		
						“..All studies had theoretical, methodological and measurement weaknesses.” p 1424		
						Cooper 1989 [57]		
Li & Jones (2013) [43]	To describe the conceptualization of nurse turnover, to evaluate the methodologies and calculation of costs in those studies, to identify the range of nurse turnover costs reported in the literature and offer suggestions for future study.	*P*	Any type of nursing staff member	1	International (by USA)	**Total number**	**10**	Nurse turnover is costly for health-care organizations, as these costs must be paid using organizational resources and accounted for in organizational budgets. The costs of per nurse turnover ranged from $10,098 to $88,000. The ratio of nurse turnover costs relative to nurses’ salary ranged from 0.31 to 1.3. The total turnover costs also ranged from $0.55 million to $8.5 million.
		*I*	Consequences	2	1990–2010	Quantitative	10	
		*C*	Not stated	3	English	*Experimental (quasi)*	*–*	
		*O*	Organisation: turnover costs			*Observational*	*10*	
		*S*	Not stated			Qualitative	**–**	
						Mix-Methods	–	
						Other	–	
						*Quality*		
						“..The scores of studies ranged between 7 and 11.” (maximum achievable 14) p.407		
						Quality index with seven criteria adapted from Beck 1995 [58]		
Schluter et al. (2008) [45]	Does unresolved moral distress and poor organizational ethical climate increase nurse turnover?	*P*	Predominantly nurses in hospital settings	1	International (by Australia)	**Total number**	**9**	There are a number of published articles characterized by loosely defined terms implying that poor ethical climate causes nurses to leave the profession. A systematic appraisal of these articles reveals that, …, it is not rigorously substantiated by the data presented
		*I*	determinants	2	1980–2007	Quantitative	6	
		*C*	Not stated	3	English	*Experimental (quasi)*	*–*	
		*O*	Nurse turnover			*Observational*	*6*	
		*S*	Qualitative / quantitative primary studies (no theoretical or discussion-based articles)			Qualitative	3	
						Mix-Methods	–	
						Other	–	
						*Quality*		
						“..Most articles were of fair quality.”p 313		
						Hawker et al. 2002 [59].		
Toh et al. (2012) [47]	The aim of this review was to establish the best available evidence regarding the relationship between the nursing shortage and nurses’ job satisfaction, stress and burnout levels in oncology/haematology settings.	*P*	RNs at inpatient and outpatient oncology/ haematology units, wards or healthcare facilities	1	International (by Singapore)	**Total number**	**7**	RNs suffered from job satisfaction, stress and burnout, which ultimately led to them leaving the specialty (oncology) or profession.
		*I*	Determinants	2	1990–2010	Quantitative	7	
		*C*	Not stated	3	English	*Experimental (quasi)*	*–*	
		*O*	Intention to leave current nursing position			*Observational*	*7*	
		*S*	Not stated			Qualitative	–	
						Mix-Methods	–	
						Other	–	
						*Quality*		
						No details available.		
						Joanna Briggs Institute Meta Analysis of Statistics Assessment and Review Instrument (JBI-MAStARI) [not referenced in Toh et al.].		
Wagner (2007) [44]	(1) What is the predictability of organizational commitment as a variable in nursing turnover studies, (2) how do organizational commitment and job satisfaction compare as predictor variables in nursing turnover studies and (3) what is the usefulness of organizational commitment in nursing turnover research?	*P*	Nurses	1	International (by USA)	**Total number**	**23**	Organizational commitment had statistically significant predictive ability in the 23 nursing turnover studies; but only 5 studies substantiated this as direct relationship. The research revealed that when using mediator variables such as intent to leave or intent to remain in turnover studies, organizational commitment is a highly desirable component. Finally, the literature demonstrated that organizational commitment is a stronger predictor of nursing turnover than is job satisfaction.
		*I*	Determinants	2	1960–2006	Quantitative	23	
		*C*	–	3	English	*Experimental (quasi)*	*–*	
		*O*	Turnover			*Observational*	*23*	
			Intent to leave or intent to remain			Qualitative	–	
		*S*	Primary studies (type not stated)			Mix-Methods	–	
						Other	–	
						*Quality*		
						No details available.		
						Not clear		

Table 2 shows each review’s criteria used to include or exclude primary studies, and the limits used to focus the reviews’ scope. The majority of the reviews limited their searches to the English language, with the exception of Flinkman et al. (2010) [11], who did not use this restriction, and Coomber and Barriball (2007) [48] who did not report this limit. The majority of the reviews did not restrict their searches by geographical region. The included reviews contained a range of seven to 31 primary studies. Of the 159 primary studies in the nine systematic reviews, 21 were included in at least two reviews, and only two primary studies [49, 50] were included in three reviews (Table 3). In the included systematic reviews, observational study designs were the most frequently reported in the included primary studies; a small number of qualitative studies were also included.

**Table 3:** Articles most frequently included in the reviews assessed

Articles		Coomber	Wagner	Schluter	Flinkman	Cowden	Toh	Chan	Li	D’Ambra
		2007 [48]	2007 [44]	2008 [45]	2010 [11]	2011 [46]	2012 [47]	2013 [41]	2013 [43]	2014 [42]
Bycio	1995 [60]		x			x				
Taunton	1997 [61]		x			x				
Ingersoll	2002 [62]		x		x					
Cowin	2002 [63]	x			x					
Lu	2002 [64]	x			x					
Larrabee	2003 [65]	x				x				
Sourdif	2004 [66]					x		x		
Lynn	2005 [67]		x		x	x				
Hart	2005 [68]			X	x			x		
Tourangeau	2006 [69]	x	x							
Chang	2006 [70]				x			x		
Estryn-Behar	2007 [71]							x		
Flinkman	2008 [72]				x			x		
Mrayyan	2008 [73]				x			x		
Chen	2008 [74]				x			x		

#### Quality assessment of included reviews

**Fig. 2:**
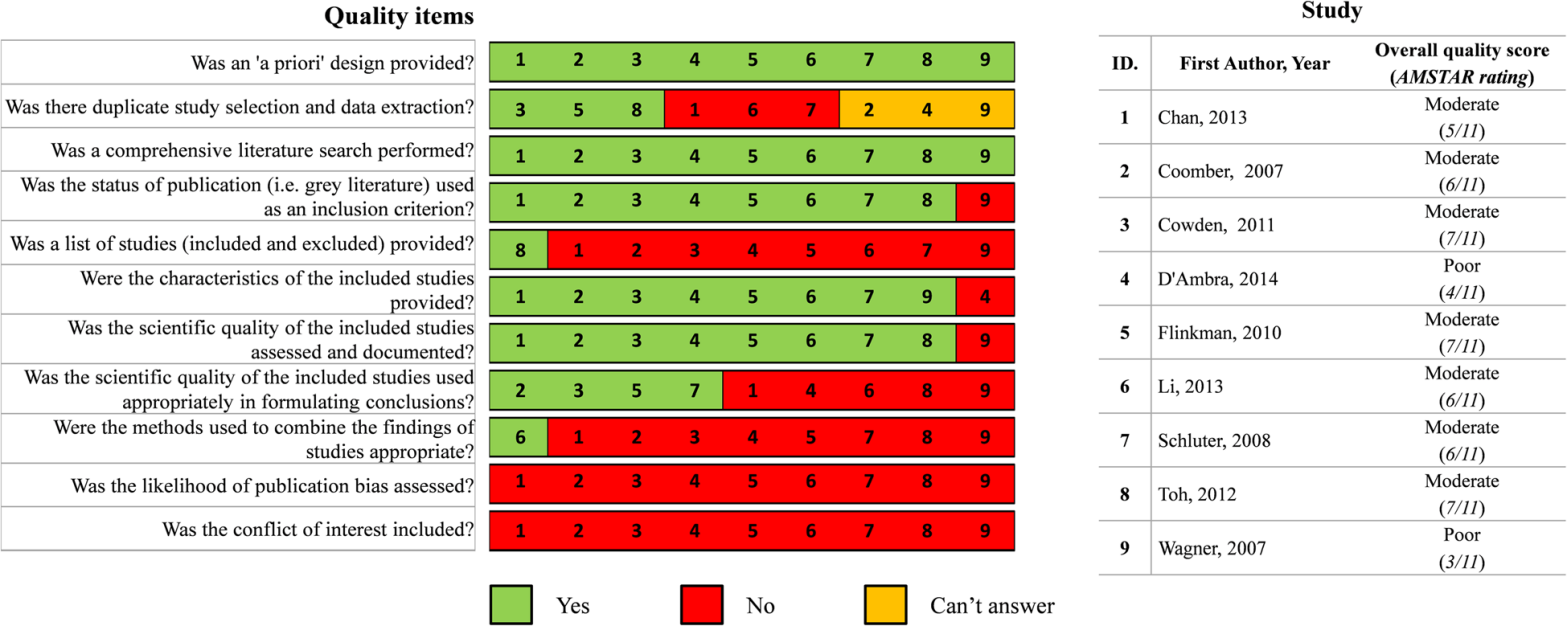
Methodological quality of the included reviews. Judgment of the presence of AMSTAR quality items in the nine reviewed reviews

Figure 2 presents the critical appraisal scores for individual reviews. The overall quality rating of the nine included systematic reviews ranged from poor (*n* = 2) [42, 44] to moderate (*n* = 7) [41, 43, 45–48].

The main reasons for reviews being in the moderate rather than strong evidence category were the lack of publication of an a priori protocol, varying levels of details about the search strategy performed, the failure to have two reviewers check the selection and data extraction, not providing a list of both included and excluded primary studies (with the exception of Toh et al. 2012 [47]), limited use of the methodological quality of included primary studies (assessed in all included reviews – the tools used to assess the quality of included papers in the included studies are shown in Table 2) and in summarising results and conclusions (used in four reviews [45, 46, 48]), and the absence of meta-analysis (or a justification for not using this method if inappropriate to the review data, apart from one review [43]).

### Results: The determinants of turnover in adult nursing

**Fig. 3:**
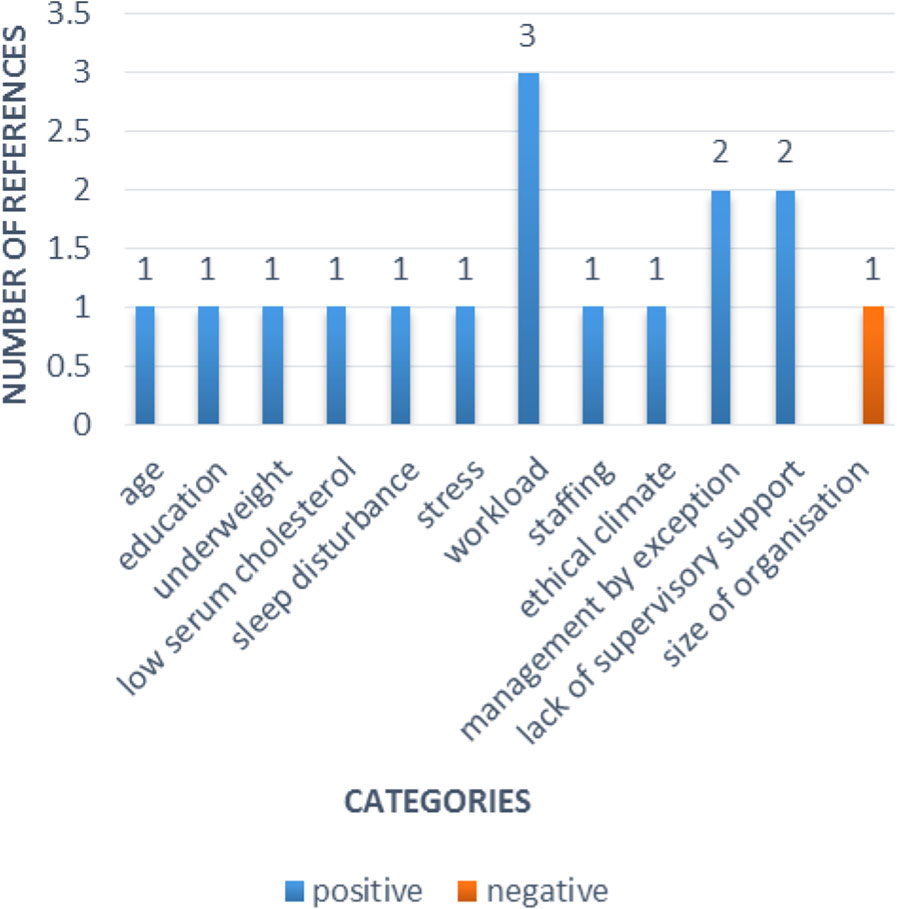
Number of primary studies per determinant for the outcome measure of turnover

**Fig. 4:**
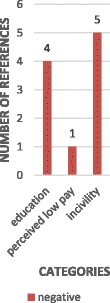
Number of primary studies per determinant reviews for the outcome measure of retention

**Fig. 5:**
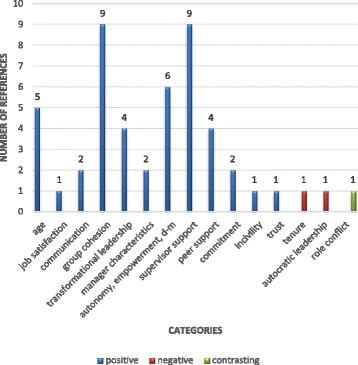
Number of primary studies per determinant for the outcome measure of intention to stay

**Fig. 6:**
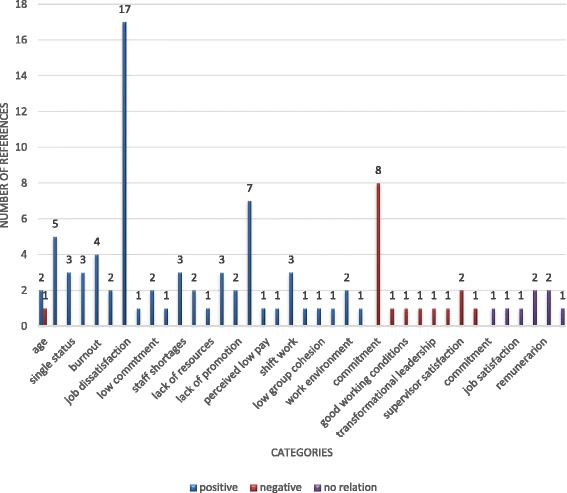
Number of primary studies per determinant for the outcome measure of intention to leave

The evidence from the included reviews is presented here by thematic analysis of determinants, grouped into four content categories: individual, job-related, interpersonal, and organisational determinants and consequences. Each of these content categories is divided by strength of evidence categories, within which we also account for the number and quality of the reviews’ included primary studies and the outcome measures reported. The four outcome measures reported were: intention to leave (in 38 primary studies), intention to stay (in 15 primary studies), turnover (in 13 primary studies) and retention (in three primary studies) (Figs. 3, 4, 5 and 6). All consequences were reported in relation to turnover.

No reviews of determinants or consequences of turnover in adult nursing were judged to be of high quality. Seven reviews were judged as of moderate quality/moderate risk of bias and addressed all four content categories of determinants. Two reviews were judged to be of poor quality.

### Individual determinants

Eleven individual determinants were reported as having been examined in five reviews of moderate quality – age, gender, marital status, educational attainment, stress, burnout, commitment, job satisfaction, low serum cholesterol, weight and sleep disturbance [41, 45, 47, 48]. Two subsets of factors were considered among individual determinants sociodemographic characteristics and psychological experiences.

The first subset of factors involved sociodemographic characteristics, some were ‘given’ characteristics such as age and gender, whereas others were acquired - education and family status. Age featured in two reviews, with contrasting findings reported. One review [41] reported an inverse relation of age and experience with intention to leave, based on splitting nurses’ age groups at 45 years or simply referring to them as ‘older’ in six quantitative primary studies, with the older group less likely to leave and nurses who had worked less than 5 years being less likely to stay. This contrasted with a positive finding of intention to leave (retire) in nurses aged over 50 from another review, [11] albeit reporting on just one primary study using a survey design, and complicated by two other studies reported by one review [41] that suggested a negative association between being a nurse aged less than 25 years and newly qualified and intention to leave and another finding a greater intention to leave in nurses older than 35 years and with longer hospital tenure (greater than 10 years) than in nurses aged under 25 years with less than a year tenure). The review authors suggested these contrasting findings to be due to the confounding of age with variables such as tenure and year post-qualification [41]. With regards to gender and marital status, one review [41] reported that male nurses and unmarried nurses had a greater intention to leave, based on three primary studies (of cross-sectional designs and excluding north American literature) for each factor. More educated nurses were reported as more likely to leave across three reviews [41, 47, 48], using different outcome measures and based on six primary studies. Chan et al.’s review (2013) [41] reported four primary studies where education was negatively associated with retention, although little detail was given on level of education; likewise there is evidence from a descriptive study of a statistically significant association between holders of master’s degrees and intention to leave their current job in specialist oncology/haematology reported by one other review’s authors [47]. Additionally, Coomber and Barriball (2007) [48] described a small but stable relationship for intention to leave with educational attainment from a meta-analysis, although when analysed with job satisfaction as an antecedent or confounding factor they report no consensus despite similar methods used in the primary studies they report and they urge caution in drawing conclusions regarding the determinant of education.

The second subset of individual characteristics described associations with psychological experiences of nurses – stress, burnout, commitment and job satisfaction. Among psychological experiences, stress and burnout are considered as negative experiences which are more likely than not to influence a decision to leave. The negative influence of stress received consistent support in three reviews [45, 48]. Two reviews reported positive associations of work-related stress (for example lack of stability in the work schedule or stress related to high workload or to the role, together with dissatisfaction of career prospects) with intention to leave [48]. These findings were based on scale-based surveys from Canada, Singapore, the UK and the USA, written comments from Australia and a meta-analysis from Taiwan, although one review [48] noted contrasting rankings of the antecedents of that stress and suggested that measurement of stress is difficult. The other review reported increased turnover [45] to be positively associated with moral stress originating in the hospital ethical climate, this definitive finding being based on one interview study, although the review authors note inferred relationships in several other studies but a lack of methodological rigour in the included studies [45]. Similarly, burnout also featured among individual factors that increased nurses’ intention to leave (including leaving the profession) in three studies of the review by Flinkman et al. (2010) [11], in one study reported by Toh et al. (2012) [47] and, alongside emotional exhaustion in a review by Chan et al. (2013) [41] reporting three different primary studies.

Job dissatisfaction or satisfaction was also reported frequently as a determinant of intention to leave or to stay. Four reviews reporting a total of 16 studies (four of which appeared in more than one review) uniformly concluded on its relationship of the measure of satisfaction/dissatisfaction used with intention to leave [41, 47, 48] or intention to stay, [41, 48] based on non-validated survey responses from a large number of nurses in studies with moderate to high response rates. One review reported no association with intention to stay [47] in responses to a survey item in one study. The sources of dissatisfaction are variously reported by the reviews from limited literature (for example nurses’ feeling dissatisfied with their inability to provide high quality of care to their patients (cited in Chan et al. 2013 [41]), dissatisfaction with staffing and workload as contributors to the intention to leave the specialty (oncology) (cited in Toh et al. 2012 [47] and dissatisfaction with salary or low pay (cited in Flinkman et al. 2010 [11]).

Commitment, presented as a positive psychological experience, featured in two reviews. One review reported a uni-directional negative relationship of organisational and occupational commitment with intention to leave the hospital [41] and another review considered different types of commitment (for example organisational, affective, continuous, normative, and professional), mostly highlighting single studies again, suggesting negative relations with intention to leave, although organisational commitment was found to have no statistical association with intention to leave nursing as a profession in one study [11]. Reviewers suggested that the multifaceted nature of commitment and different designs and tools impact on findings. One further review, [44] judged to be of poor quality, contributed mixed evidence regarding commitment as a determinant, describing 12 studies with negative associations with intention to leave and two studies with significant negative associations with turnover, as well as two other studies confirming a positive influence of organisational commitment on intention to stay.

Additionally, the impact of biological factors (low serum cholesterol, being underweight, sleep disturbance) on intention to leave is considered in one review, [41] relying on a single study for this evidence.

### Job-related determinants

Three reviews synthesised evidence around seven job-related and occupational determinants – work content, workload, task variation, role ambiguity, shift patterns, rota stability and promotional opportunities. Workload, including demanding work content, high workload, variation in work tasks or role ambiguity were reported to increase intention to leave in one study and turnover in two others, while one study found no association with intention to leave [41]. Working patterns, such as shift work (evenings and night shifts mentioned specifically) [41] were linked to intention to leave, and increasing stability from a constantly changing rota as a way to reduce stress [48] was reported as negatively associated with intention to leave. Promotional opportunities featured an influential factor too. Intention to leave increased where nurses experienced fewer possibilities for development or professional growth, evidenced by two studies in one review [11] and four studies (one overlapping) of another review, including the findings of a large study carried out in 10 European countries [41]. Chan et al. (2013) [41] also cited three quantitative studies confirming the impact of lack of autonomy on intention to leave. Role conflict has also been suggested to be a determining factor in decreasing a nurse’s intention to stay in one study in one review, [41] while another review [11] reported a study providing conflicting quantitative and qualitative findings from the same group of nurses; this review suggested that more experienced nurses (how they saw themselves professionally) indicated an intention to stay.

### Interpersonal determinants

The evidence on the impact of interpersonal factors included the consideration of ten determinants related to supervisor support; managerial style – praise and recognition, trust, manager characteristics; leadership practices; staff autonomy, empowerment and decision making; group cohesion; social support; team work and workplace incivility.

Supervisor support featured in two reviews, [46, 48] with a total of 15 primary studies stating, relatively unambiguously, that this had a positive influence on intention to stay, with just one primary study cited as an exception in Coomber and Barriball (2007) [48]. This association was illustrated by direct and indirect associations (for example, via empowerment in one study cited in one review [48]. Along the same lines, satisfaction with a supervisor was reported as negatively related to intention to quit in one study in one review [48].

Additionally, the positive influence of praise and recognition and of trust in manager was significantly correlated with intent to stay (each characteristic evidenced by singular studies in one review [46]). Broadly defined ‘poor management’ featured in a qualitative study as positively related to intention to leave [41].

With regards to types of leadership the reviews revealed that transformational (and generally participative) managerial style increased intention to stay [46] or decreased intention to leave (although the relationship was through other factors) [48]. On the contrary, the transactional leadership style of ‘management by exception’, whereby managers only act on deviations from plan or budget, was found to increase turnover rates, and autocratic leadership was significantly negatively correlated with intention to stay [46]. However, some of the specific manager’s characteristics, in particular, the degree of power and influence the nurse perceived their manager to have within an organisation, received significantly positive association with the intention to stay [46].

The positive and significant influences of empowerment, control over practice and shared decision-making on intent to stay received support in six studies reported in one review [46]. Group cohesion also appeared to be important with nine studies reported in the same review [46] showing a significant positive relationship with intent to stay in the current nursing position. In a similar vein, the review by Chan et al. (2011) [41] contained a few references to the importance of social support and good communication with supervisors for nurses’ intention to stay, particularly, in a hospital. Low quality teamwork, on the opposite, was said to be associated with higher intention to leave [41].

These consistent findings across a number of studies in the three reviews are tempered somewhat by the review authors’ comments arising from their quality appraisal of the evidence. For example, Cowden et al. (2011) [46] raised some concern over biases of synthesis such as over-reporting of positive findings, and lack of causal analysis between leadership factors, as well as the limits to generalisability imposed by heterogeneous studies, this point also being relevant for Coomber and Barriball (2007) [48] who noted a heavy reliance on mixed samples and scales.

One relatively stand-alone review judged to be of poor quality in our overview looked at an interpersonal determinant workplace incivility, in particular, behaviours violating workplace standards and consideration towards new graduate nurses [42]. Lateral violence, that is co-workers’ violence that redirects aggression towards those in authority on their more vulnerable co-workers was reported as a major factor in the decision to leave nursing by 14% of RNs in a survey study and its indirect effect on low retention in new graduates was reported across five other studies. Assessment of rigour and quality in this particular review is however impeded by missing information on the characteristics of the included studies.

### Organisational determinants

Seven organisational factors outlined three strands of evidence: work environment including climate, organisational structure and financial determinants.

One review [41] cited three studies that demonstrated the influence of work environment, for example, the perceptions of a ‘deteriorated external work environment’ as increasing intention to leave, and ‘better working conditions’ as lowering it; however these concepts were not defined. This review also contained reference to ethical climate as a key aspect of work environment that can significantly influence the turnover intentions of registered nurses, referencing the same single, though robust, study as in one other review [45]. Limited evidence was found on the impact of organisational culture, with one review suggesting from two studies of Asian nurses in Asian countries that the individualism-collectivism dichotomy could relate to turnover phenomena: a collectivistic cultural factor played an important role in weakening nurses’ intention to leave [41].

The influence of staff shortages as well as lack of resources on intention to leave was mentioned from one qualitative study where the shortage of nurses implied insufficient manpower to satisfy nurses’ personal standards of care, and one questionnaire study focused on patient workloads in one review [41]. Conversely, a single study cited in the same review [41] also suggested that working in smaller outpatient and day care units generated a negative association with turnover.

Another set of organisational determinants was that of financial incentives. One review [41] listed six primary studies suggesting that those nurses dissatisfied with their remuneration were more likely to leave, and that social rewards such as pay and job security were ranked higher for some generations (born 1946–1959) than others. Gender was highlighted by another review [48] with male participants reported in one study as being twice as likely in their intention to leave as females due to dissatisfaction with salary. The results of other three studies reviewed in one review [48], produced from differing methods of assessment, suggested non-uniform relations between pay and retention. Although factor analysis showed pay as an important contributor to job satisfaction, pay was not a statistically significant indicator of intent to leave or turnover cognition. Written comments from two studies conducted in Australia and USA indicated that fairness and equality of pay was more important to nurses in retaining their positions. In other words, perceived low pay had a greater influence than pay level per se. Crucial factors were commensuration according to contributions, for example, for roles with high responsibility, and additional reward mechanisms including fringe benefits [48].

### Findings on the consequences of turnover in adult nursing

Only one review included evidence of the consequences of turnover, [43] and this review was judged to be of moderate quality / moderate risk of bias. This review focused solely on cost as the consequence of turnover [43]. This review was based on ten studies, eight of which were in acute hospital settings, all conducted in the USA, with one also in each of Australasia and Canada. The review reported costs of per nurse turnover ranging from $10,098 to $88,000 and a total turnover cost ranging from $0.55 million to $8.5 million, the ratio of nurse turnover costs relative to nurses’ salary ranging from 0.31 to 1.3. Orientating and training new hires was reported as the largest or second largest category of costs relative to total nurse turnover costs while several studies also noted the high costs of unfilled positions/vacancy costs (defined usually as the costs of temporary replacements, but also including wider costs, for example, patient deferral costs and productivity costs for supervisors and other staff, in some primary studies they review). The review authors note the difficulty interpreting and generalising from their included primary studies due to the variability in conceptualisation and measurement of turnover, in time-periods (spanning over two decades) and geographic locations. They also noted that all but one study, which was based on econometric methods, relied on descriptive statistical analyses and that the studies were mostly based in one setting and had relatively small sample sizes. That said the key message from the review was that nurse turnover is costly for organisations.

## Discussion

### Summary of findings from and limitations of the included reviews

Our overview (review of systematic reviews) points us to a complex range of determinants of turnover in adult nursing, at the individual, job-related, interpersonal and organisational level, and to the cost consequences of turnover, but many reviews only cite one or two primary studies for many of the determinants they feature. The analysis here reveals that despite the publication of a large number of primary studies (*n* = 159 in the reviews of primary studies we reported fully in the narrative of reviews), there is a low degree of overlap in their presence in eight reviews which focus on the same topic and present similar categories of determinants. We might suggest that the low overlap could be attributed to differences in the detail of the research questions (for example, concentrating on job satisfaction [48] or commitment; [44] see Table 2) as the international reviews with more general research questions have a greater overlap [41]. Nevertheless, the impact of this is a rather disjointed body of evidence in which both the outcome of actual turnover as opposed to intention to leave is poorly addressed, and modelling of determinants in combination, taking account of confounding factors, is rare. While the large number of reviews on the topic of nurse turnover may give the impression that the topic is saturated, our overview suggests new knowledge -that there are large gaps in the literature on determinants of turnover in adult nursing. Review of the literature on the consequences of determinants is rare, although we note that some conflate these issues as consequences such as reduced staff numbers are also related to determinants such as workload pressures.

The most strongly supported determinants of turnover in the literature reviewed were at the individual level: stress and burnout, job dissatisfaction and (to a lesser degree) commitment. Supervisor support was the most supported determinant for retention.

The reviews use a number of outcome measures - intention to leave, turnover, intention to remain and retention – and many present these unquestioningly as measuring the same concept. The largest number of reviews uses the measures of intention, in particular, intention to leave, rather than action. This is problematic as, although intention has been demonstrated to be a consistent predictor of nurse retention, how these behavioural intentions develop and the link between intention to leave a job and actually leaving are unclear [46]. Furthermore, the inconsistency in the criteria and outcomes measures used in research studies and reviews not only demonstrates the complexity of the concept of turnover, it also shows how reviews of the turnover evidence have not systematically built on previous work in a consistent way to contribute to a shared theoretical base, despite discussion about definitions, conceptual models and a need for multivariable analyses [10]. Concepts therefore remain loosely defined and are used interchangeably. It might be that this accounts for the very limited evidence related to consequences at organisational level (cost), with no evidence on individual level consequences.

The quality of the reviews was mostly moderate, and, while all nine reviews stated that they had carried out a quality appraisal of their included primary studies, only one of the reviews used the assessment of studies to support their reporting and conclusions; however we know that the primary studies they report are predominantly quantitative observational designs, most often based on self-report data, with a small number of qualitative studies also included. More positively, several of the reviews highlight limitations of the body of literature, such as poor definition of intention to leave, dependence on cross sectional survey designs (with qualitative investigative depth mostly lacking [48]) and variability in the health systems of different countries in particular (identified in two reviews [41]), as well as noting the emphasis on single studies in several reviews [11], and the heterogeneity of nurses, [11] often within studies [48]. Difficulties comparing across reviews due to other issues of definition, for example, of moral climate [45] or definition and measurement of manager leadership practices [46] or poor specificity of workplaces studied [47] are also raised. The limitations associated with meta analysis being prevented by the above mentioned heterogeneity are also specifically mentioned [46]. This degree of critique can be considered to ameliorate some reviews within the grouping of moderate strength of evidence in particular.

### Limitations and strengths of our overview

Our overview is limited by design. In being an overview of (systematic) reviews we have relied upon the review authors’ reporting and interpretation of the primary studies and have made some assumptions about quality based on descriptions of research design rather than on a critical appraisal of each primary study. We suggest that this limitation is mitigated somewhat by only including reviews that have at least reported that they have carried out a quality appraisal of their included studies, and becomes a strength in that we have sought to review rather than add yet another review of primary studies to the large, somewhat repetitive, yet also heterogeneous decades of literature on turnover in adult nursing. We have also assessed the quality of the included reviews using a widely recognised tool for this task [37]. Our decision to include those reviews that reported a quality appraisal of their included studies also limits our review in excluding from our full narrative a number of comprehensive and recent reviews of the determinants and consequences of turnover in adult nursing that added considerably to our thematic index. In particular we have not featured the national/societal or patient level determinants and patient care outcomes that appear in the twelve reviews that did not contain a methodological appraisal of their included primary studies although they met our other inclusion criteria. We may also have excluded high quality primary studies that did not feature in reviews containing a quality appraisal. While this is acknowledged here as a limitation, we however consider this justified, and indeed a strength of our overview, in that we have based this decision on the guidance for the good conduct of systematic reviews available since the 1990s [26] and have only included reviews published since that date. We have therefore provided a focused account of what should be the highest quality reviews available. In spite of this, our own overview is limited in the conclusions it can come to regarding the determinants and consequences of turnover by the limitations of the systematic reviews that we systematically reviewed, for two reasons in particular. First, the coterminous use of outcome measures of intention with those of action (that is intention to leave with turnover, for example) is problematic and we are also limited in that we have partially replicated this concern in this overview, whilst also seeking to be explicit about the measures we have combined. This issue is considered in-depth in the turnover literature outside of nursing with acknowledgment of the poor translation of intentions to behavior [25] illustrated through wide statistical credibility estimates of the relationship [23]. Evidence suggests that the relationship can be moderated by, for example, structural variables [25] or personality traits [49]. As intentions are considered to overestimate actual performance (here, actual turnover), the determinants we present may have moderator effects not previously presented in the nursing literature. An important recommendation of this review is that the concepts related to nursing turnover are carefully considered and defined and consensus reached about the priorities for future research and workforce development to increase the pertinence and co-ordination of future research to provide evidence that can inform decision making in human resources practice and planning in healthcare and nursing. Second, and fundamentally, we are limited by the absence of any reviews that have been assessed to offer strong evidence. The literature we reviewed offered no opportunities to carry out the meta-analysis of antecedents and correlates which we find in the broader human resource turnover literature, where not only are primary studies’ findings statistically pooled, but variations in base rates of turnover and moderators in statistical models of turnover are tested [23]. We also note that the majority of the reviews we included did not specify what type of ‘leaving’ their primary studies referred to, that is leaving a department, an employer or the profession; only four of the studies mention this; three of these refer to leaving the profession.

Finally, with the inevitable time lag of publication of primary studies to their inclusion in a pertinent review, we are likely to have missed all of the more recent literature published.

### Our findings in the context of other literature

From our searches we identified 66 reviews already published on this topic, including recent developments in conceptualising the determinants and consequences of such turnover into models [28]. However, when we applied criteria based upon guidance for the good conduct of systematic reviews [26] we systematically and explicitly excluded large numbers of reviews, and reviewed a relatively small number in full. The results are not surprising in content of determinants and consequences as we developed a thematic index based on the reviews we were reviewing, several of which grouped determinants similarly, for example using the groupings of individual, interpersonal and organisational factors [27]. The results are also not entirely surprising when viewed in the context of the broader management literature on the wide range of researched antecedents to turnover – for example, if we look at what Holtom et al. (2008) [24] described as the major trends in turnover research in the preceding decade, our overview points to some evidence on the role of interpersonal relationships, of organisational commitment and embeddedness and of job satisfaction, but it does not present evidence in the nursing literature on individual difference predictions such as personality or of working conditions; nor of dynamic processes. The overview also contains substantial literature related to demographic issues that Griffeth et al. (2000) [23] consider to be decreasing in importance. The rising issues of social networks [25] and cultural differences [24], as well as multi-level investigations [24] are equally lacking in visibility in the reviews we have included. In recognition of these differences, and the limitations of the quality of the literature and the predominance of intention to leave versus actual turnover in the nursing turnover literature, we have not sought to try to fit it to one particular model from the literature outside of nursing.

It is in recognition of the plethora of previous work in nursing that we conducted this overview of systematic reviews and, in doing so, highlight an important finding: while clarity has been achieved on where the strongest current evidence lies regarding the determinants and consequences of adult nursing turnover, none of the evidence is strong when we combine different interventions, different outcomes, different conditions, problems or populations, as suggested for reviews of reviews [50]. Despite the plethora of reviews, the gaps in strongly evidence-based knowledge about adult nursing turnover limit the conclusions that can be drawn even from the relatively stronger reviews from which we built our overview. We suggest that this could contribute to a continuing problem, if managerial decision makers have not been clearly signposted to robustly conducted systematic reviews based on robustly conducted and/or robustly critiqued primary studies.

## Conclusions

The current evidence is incomplete and has a number of important limitations. A body of moderate quality review evidence does exist giving a picture of multiple determinants of turnover in adult nursing, with individual level nurse stress and dissatisfaction factors and organisational level managerial style and supervisory support factors holding most weight, as well as the economic consequence of the turnover. Our systematic review of the review literature uses the quality of the review alongside the quality of the included primary studies and which outcomes they measure to progress the usefulness of the body of review literature for decision makers, in terms of the determinants themselves. In using the quality of the review alongside the quality of the included primary studies and which outcomes they measure the evidence is far from definitive. Further research, of rigorous research design, drawing on recommendations from the wider management literature on turnover, whether quantitative or qualitative, particularly against the outcome of actual turnover as opposed to intention to leave, and modelling determinants in combination, taking account of confounding factors, is required.

## Additional files

Additional file 1: Turnover in adult nursing: OVERVIEW: Search strategies for individual databases on determinants and consequences. (XLS 80 kb)

Additional file 2: Turnover in adult nursing r OVERVIEW: Thematic index of determinants and consequences. (DOCX 22 kb)

Additional file 3: Turnover in adult nursing OVERVIEW: Content and thematic analysis. (XLSX 26 kb)

Additional file 4: Turnover in adult nursing OVERVIEW: Excluded studies on determinants and consequences. (DOCX 65 kb)

## Abbreviations

AMSTAR: Assessment of Multiple Systematic Reviews
C: Comparison
I: Intervention
O: Outcome
P: Population
PICOS: Population, Intervention, Comparison, Outcomes, Study design
RN: Registered nurse
S: Study design
