# Supplementary material for: The determinants and consequences of adult nursing staff turnover: a systematic review of systematic reviews
Source: BMC Health Serv Res. 2017 Dec 15;17:824. doi: 10.1186/s12913-017-2707-0 (PMC5732502; doi:10.1186/s12913-017-2707-0)
Supplement: Supplementary file 2 — Turnover in adult nursing r OVERVIEW: Thematic index of determinants and consequences. (DOCX 22 kb) [file 12913_2017_2707_MOESM2_ESM.docx]

**Turnover in adult nursing OVERVIEW**

**THEMATIC INDEX_determinants and consequences**

**I. Index of determinants**

1. Individual factors
   1. Sociodemographic characteristics
      1. Given
         1. Age (at least three intervals)
         2. Gender
      2. Acquired
         1. Education
         2. Family status, kinship
         3. Tenure
      3. Biological
         1. Low serum cholesterol
         2. Underweight
         3. Sleep disturbance
   2. Psychological experiences
      1. Negative experiences
         1. Burnout/ Emotional exhaustion
         2. Stress / Moral distress or sensitivity
         3. Job dissatisfaction
      2. Positive experiences
         1. Commitment (affective, normative, continuance)
         2. Respect, recognition
         3. Job satisfaction
2. Interpersonal
   1. Leadership practices
   2. Management style
      1. Manager characteristics
      2. Communication
      3. Staff autonomy, empowerment and decision-making
   3. Supervisor support
   4. Peer relations
      1. Positive
         1. Group cohesion
         2. Teamwork
         3. Support
      2. Negative
         1. Incivility
3. Job/professional
   1. Positive aspects
      1. Flexibility (shift work, working hours, variations in work tasks)
      2. Promotional opportunities
      3. Employment opportunities
   2. Negative aspects
      1. Lack of resources
      2. Workload including physical demands
      3. Role conflict
4. Organisational
   1. Structural
      1. Remuneration, pay and benefits
      2. Size
      3. Speciality
   2. Organisational culture
      1. Cultural
      2. Ethical climate (including incivility)
      3. Work environment, otherwise unspecified
5. System-level
   1. Societal
      1. Perception of nursing
      2. Generation Y
   2. National
      1. Labour supply
      2. Shortages
      3. Economic downturn
6. Patient-related
   1. Patient satisfaction
   2. Quality and safety of care (hospitalisation, risk of infection, mortality, adversities, etc).

**II. Index of consequences**

1. Costs
2. Staffing levels, shortage and impact on quality of care
3. Patient outcomes
4. Morale
