# Supplementary material for: The determinants and consequences of adult nursing staff turnover: a systematic review of systematic reviews
Source: BMC Health Serv Res. 2017 Dec 15;17:824. doi: 10.1186/s12913-017-2707-0 (PMC5732502; doi:10.1186/s12913-017-2707-0)
Supplement: Supplementary file 4 — Turnover in adult nursing OVERVIEW: Excluded studies on determinants and consequences. (DOCX 65 kb) [file 12913_2017_2707_MOESM4_ESM.docx]

**Turnover in adult nursing OVERVIEW**

**Excluded studies_Determinants and consequences**

[Part 1: Full-text articles excluded, with reasons 1](#_Toc426128195)

[Part 2: General characteristics of the excluded reviews (those not including a quality assessment of the included papers) 5](#_Toc426128196)

# Part 1: Full-text articles excluded, with reasons

| **Reason for exclusion** | **References of excluding studies** |
| --- | --- |
| **Not intervention of interest** | Please contact the corresponding author for details |
| **Any review using informal and subjective methods to collect and interpret evidence** | 1. Almost J. Review: The consequences of executive turnover. Journal of Research in Nursing. 2011;16(6):515-7. 2. Andrews DR, Dziegielewski SF. The nurse manager: Job satisfaction, the nursing shortage and retention. J Nurs Manag. 2005;13(4):286-95. 3. Bingham R. Leaving nursing. Health Aff. 2002;21(1):211-7. 4. Buchan J. O’May F. Nursing supply and demand: Reviewing the evidence. Nursing Times 1998; 94(26):60-63. 5. Cowden TL, Cummings GG. Nursing theory and concept development: A theoretical model of clinical nurses' intentions to stay in their current positions. J Adv Nurs. 2012;68(7):1646-57. 6. Crow SM, Hartman SJ. Nurse attrition as a process. Health Care Manag. 2005;24(3):276-83. 7. Curtin LL. An integrated analysis of nurse staffing and related variables: Effects on patient outcomes. Online Journal of Issues in Nursing. 2003;8(3):59-71. 8. Day GE, Minichiello V, Madison J. Nursing morale: what does the literature reveal? Aust Health Rev. 2006 Nov;30(4):516-24. 9. Dowswell T, Hewison J, Millar B. Enrolled nurse conversion: trapped into training. Journal of Advanced Nursing. 1998;28:540–547. 10. Ehlers VJ. 'Professional nurses' requests to remove their names from the South African Nursing Council's register. Part 1: Introduction and literature review', Health SA Gesondheid 2003; 8(2), 63-69. 11. Ellenbecker CH. A theoretical model of job retention for home health care nurses. J Adv Nurs. 2004;47(3):303-10. 12. Ferguson LM, Day RA. Challenges for new nurses in evidence-based practice. J Nurs Manag. 2007;15(1):107-13. 13. Fisher KA, Fraser JD. Rural health career pathways: Research themes in recruitment and retention. Australian Health Review. 2010;34(3):292-6. 14. Heinz D. Hospital nurse staffing and patient outcomes: a review of current literature. Dimensions of critical care nursing : DCCN. 2004;23(1):44-50. 15. Henry JD, Henry LS. Living and leaving your nursing legacy. Okla Nurse. 2006;51(1):17. 16. Hogan TD. The impact of staff case manager-case management supervisor relationship on job satisfaction and retention of RN case managers. Lippincott's case management : managing the process of patient care. 2005;10(5):246-53. 17. Jackson D, Mannix J, Daly J. Retaining a viable workforce: a critical challenge for nursing. Contemporary Nurse. 2001;11:163–172. 18. Janiszewski Goodin H. The nursing shortage in the United States of America: An integrative review of the literature. J Adv Nurs. 2003;43(4):335-43. 19. Kleinman CS. Leadership and Retention: Research Needed. J Nurs Adm. 2004;34(3):111-3. 20. Manahan C, Lavoie JG. Who stays in rural nursing practice? An international review of the literature on factors influencing rural nurse retention. Online J Rural Nurs Health Care. 2009;9:82–93. 21. Mills J, Birks M, Hegney D. The status of rural nursing in Australia: 12 years on. Collegian. 2010;17(1):30-7. 22. Montenery S, Shearer J, Johnson S. Nursing professional development: stories, tips, and techniques. Journal for nurses in staff development : JNSD : official journal of the National Nursing Staff Development Organization. 2012;28(5):250-2. 23. Moseley A, Jeffers L, Paterson J. The retention of the older nursing workforce: A literature review exploring factors that influence the retention and turnover of older nurses. Contemporary Nurse. 2008;30(1):46-56. 24. O'Brien-Pallas L, Hayes L. Challenges in getting workforce research in nursing used for decision-making in policy and practice: A Canadian perspective. J Clin Nurs. 2008;17(24):3338-46. 25. Stamler LL, Gabriel AM. Professional failure to thrive: a threat to high-quality care? Nursing leadership (Toronto, Ont.). 2010;23(1):22-31. 26. Steel RP, Lounsbury JW. Turnover process models: Review and synthesis of a conceptual literature. Human Resource Management Review. 2009;19(4):271-82. 27. Stone PW, Tourangeau AE, Duffield CM, Hughes F, Jones CB, O’Brien-Pallas, L. et al, Evidence of nurse working conditions(a global perspective) . Pol Politics Nurs Practice. 2003;4:120–130. 28. Takase M, Maude P, Manias E. Nurses' job dissatisfaction and turnover intention: Methodological myths and an alternative approach. Nursing and Health Sciences. 2005;7(3):209-17. |
| **Inappropriate study design (i.e. no literature review; reports from any type of primary study)** | 1. Atwater A, Hartmann E, Brown BB, Carteaux P, Freeman M, Hegwood P, et al. Evaluation and development of potentially better practices for staffing in neonatal intensive care units. Pediatrics. 2006;118(SUPPL. 2):S134-40. 2. Brunetto Y, Xerri M, Shriberg A, Farr-Wharton R, Shacklock K, Newman S, et al. The impact of workplace relationships on engagement, well-being, commitment and turnover f or nurses in Australia and the USA. J Adv Nurs. 2013;69(12):2786-99. 3. Buchan J. Still attractive after all these years? Magnet hospitals in a changing health care environment. J Adv Nurs. 1999 Jul;30(1):100-8. 4. Dingley J, Yoder L. The public health nursing work environment: Review of the research literature. Journal of Public Health Management and Practice. 2013;19(4):308-21. 5. Erenstein CF, McCaffrey R. How healthcare work environments influence nurse retention. Holist Nurs Pract. 2007;21(6):303-7. 6. Heilmann P. To have and to hold: personnel shortage in a Finnish healthcare organisation. Scand J Public Health. 2010;38(5):518-23. 7. Kirkwood M, Wales A, Wilson A. A Delphi study to determine nursing research priorities in the North Glasgow University Hospitals NHS Trust and the corresponding evidence base. Health information and libraries journal. 2003;20 Suppl 1:53-8. 8. Lambert VA, Lambert CE, Petrini M, Li XM, Zhang YJ. Predictors of physical and mental health in hospital nurses within the People's Republic of China: Original article.Int Nurs Rev. 2007;54(1):85-91. 9. Muncey T. Selection and retention of nurses. J Adv Nurs. 1998;27(2):406-13. 10. Nancarrow S. The impact of intermediate care services on job satisfaction, skills and career development opportunities. J Clin Nurs. 2007;16(7):1222-9. 11. Newman K, Maylor U, Chansarkar B. The nurse retention, quality of care and patient satisfaction chain. Int J Health Care Qual Assur. 2001;14(2):57-68. 12. Price SL, Mcgillis Hall L. The history of nurse imagery and the implications for recruitment: A discussion paper. J Adv Nurs. 2014;70(7):1502-9. 13. Reitz OE, Anderson MA. An Overview of Job Embeddedness. Journal of Professional Nursing. 2011;27(5):320-7. 14. Ritter D. The relationship between healthy work environments and retention of nurses in a hospital setting. J Nurs Manag. 2011;19(1):27-32. 15. Scott S, Huntington A, Baker H, Dickinson A. The graduate nursing workforce: does an international perspective have relevance for New Zealand? Nurs Prax N Z. 2011;27(3):4-12. 16. Serratt T. California's nurse-to-patient ratios, Part 1: 8 years later, what do we know about nurse-level outcome? J Nurs Adm. 2013 Sep;43(9):475-80. doi: 10.1097/NNA.0b013e3182a23d6f. 17. Smith HL, Hood JN, Waldman JD, Smith VL. Creating a favorable practice environment for nurses. J Nurs Adm. 2005;35(12):525-32. 18. Strachota E, Normandin P, O'Brien N, Clary M, Krukow B. Reasons registered nurses leave or change employment status. J Nurs Adm. 2003;33(2):111-7. 19. Takase M. A concept analysis of turnover intention: Implications for nursing management. Collegian. 2010;17(1):3-12. 20. Temple A, Dobbs D, Andel R. Exploring correlates of turnover among nursing assistants in the National Nursing Home Survey. J Nurs Adm. 2011;41(7-8 SUPPL.):S34-42. |
| **Non peer-reviewed articles (i.e. thesis, conference papers, book chapters; reviews published only in abstract form)** | 1. Barlow KM. A Meta-Analysis of Transformational Leadership and Subordinate Nursing Personnel Organizational Commitment, Job satisfaction, and Turnover Intentions. Ph.D Dissertation., UNIVERSITY OF MARYLAND, BALTIMORE, 2013, 291 pages; 3563301 2. Buchan J. Nurses' turnover: reviewing the evidence, heeding the results? J Adv Nurs. 2013 Sep;69(9):1917-8. doi: 10.1111/j.1365-2648.2012.06080.x. Epub 2012 Aug 5. 3. Djukic M. Review: Job satisfaction and intentions to leave of new nurses. Journal of Research in Nursing. 2011;16(6):549-50. 4. Gibbs I, McCaughan D, Griffiths M. Skill mix in nursing: a selective review of the literature. J Adv Nurs. 1991;16(2):242-9. 5. Irvine DM, Evans MG. Job satisfaction and turnover among nurses: integrating research findings across studies. Nurs Res. 1995;44 (4):246-53. 6. Jennifer L. Kettle RN Factors Affecting Job Satisfaction in the Registered Nurse 1987 Aug Res Nurs Health 10(4):253-61 7. Kleinpell R. Evidence-based review and discussion points. American Journal of Critical Care. 2010;19(5):441-2. 8. MacWilliams BR, Schmidt B, Bleich MR. Men in nursing. Am J Nurs. 2013 Jan;113(1):38-44; quiz 45. doi: 10.1097/01.NAJ.0000425746.83731.16. 9. MacWilliams BR, Schmidt B, Bleich MR. Men in nursing: Understanding the challenges men face working in this predominantly female profession. Am J Nurs. 2013;113(1):38-44. 10. Meadows S, Levenson R, Baeza J. The last straw: explaining the NHS nursing shortage. London: King's Fund; 2000. 11. Positive Working Relationships Matter for Better Nurse and Patient Outcomes. Spence Laschinger HK, ed. J Nurs Manag. 2010;18:875-1086. 12. Tucker K. Heward Y.2014, 'What factors might affect PIC nurse retention? A review of the literature.'. Paper presented at the 7th World Congress on Pediatric Intensive and Critical Care, PICC 2014 Istanbul Turkey 13. Walton N. Review: What factors affect nursing retention in the acute care setting? Journal of Research in Nursing 2011; 16:501-502. |
| **Reviews not including a quality assessment of the included papers**  ***(please see part 2 of this file for further details)*** | 1. Alameddine M, Baumann A, Laporte A, Deber R. A narrative review on the effect of economic downturns on the nursing labour market: Implications for policy and planning. Human Resources for Health. 2012;10. 2. Borda RG, Norman IJ. Factors influencing turnover and absence of nurses : A research review. Int J Nurs Stud. 1997;34(6):385-94. 3. Daouk-Öyry L, Anouze A-, Otaki F, Dumit NY, Osman I. The JOINT model of nurse absenteeism and turnover: A systematic review. Int J Nurs Stud. 2014;51(1):93-110. 4. Duffield CM, Roche MA, Homer C, Buchan J, Dimitrelis S. A comparative review of nurse turnover rates and costs across countries. J Adv Nurs. 2014;70(12):2703-12. 5. Duvall JJ, Andrews DR. Using a Structured Review of the Literature to Identify Key Factors Associated With the Current Nursing Shortage. Journal of Professional Nursing. 2010;26(5):309-17. 6. Ellenbecker CH, Cushman M. Home healthcare nurse retention and patient outcome model: Discussion and model development. J Adv Nurs. 2012;68(8):1881-93. 7. Hayes LJ, O'Brien-Pallas L, Duffield C, Shamian J, Buchan J, Hughes F, et al. Nurse turnover: A literature review - An update. Int J Nurs Stud. 2012;49(7):887-905. 8. Hayes LJ, O'Brien-Pallas L, Duffield C, Shamian J, Buchan J, Hughes F, et al. Nurse turnover: A literature review. Int J Nurs Stud. 2006;43(2):237-63. 9. Hutchinson D, Brown J, Longworth K. Attracting and maintaining the Y Generation in nursing: A literature review. J Nurs Manag. 2012;20(4):444-50. 10. Lim J, Bogossian F, Ahern K. Stress and coping in Singaporean nurses: A literature review. Nursing and Health Sciences. 2010;12(2):251-8. 11. Storey C, Cheater F, Ford J, Leese B. Retention of nurses in the primary and community care workforce after the age of 50 years: Database analysis and literature review. J Adv Nurs. 2009;65(8):1596-605. 12. Wai Chi Tai T, Bame SI, Robinson CD. Review of nursing turnover research, 1977-1996. Social Science and Medicine. 1998;47(12):1905-24. |
| **Inappropriate study population** | 1. Brown P, Fraser K, Wong CA, Muise M, Cummings G. Factors influencing intentions to stay and retention of nurse managers: A systematic review. J Nurs Manag. 2013;21(3):459-72. 2. Morgan D. Caring for dying children: assessing the needs of the pediatric palliative care nurse. Pediatr Nurs. 2009;35(2):86-90. 3. McVicar A. Workplace stress in nursing: A literature review. J Adv Nurs. 2003;44(6):633-42. 4. "Pearson A, Porritt K, Doran D, Vincent L, Craig D, Tucker D, Long L. A systematic review of evidence on the professional practice of the nurse and developing and sustaining a healthy work environment in healthcare. Int J Evid Based Healthc. 2006 Sep;4(3):221-61. doi: 10.1111/j.1479-6988.2006.00046.x. |
| **Reviews published before 1990** | 1. Cavanagh SJ. Predictors of nursing staff turnover. J Adv Nurs. 1990;15(3):373-80. 2. Cavanagh SJ. Nursing turnover: literature review and methodological critique. J Adv Nurs. 1989;14(7):587-96. 3. Buckle P. Epidemiological aspects of back pain within the nursing profession. Int J Nurs Stud. 1987;24(4):319-24. |
| **No English** | 1. Yin JC, Yang KP. Nursing turnover in Taiwan: a meta-analysis of related factors. Int J Nurs Stud. 2002 Aug;39(6):573-81. |

# Part 2: General characteristics of the excluded reviews (those not including a quality assessment of the included papers)

| **First Author**  **year** | **Aim(s)**  **Research question(s)** | **Selection criteria used to include primary studies (PICOS)** | **Scope**  1. Geography  2. Time limit  3. Language | **Type, number, and quality of included studies** **as reported by the author(s)** | **Review authors’**  **summary of findings** | **AMSTAR score** |
| --- | --- | --- | --- | --- | --- | --- |
| Alameddine  2012 | To examine the effects of economic downturns and recession on the supply and demand of nurses and provide a framework to help healthcare decision makers understand and manage their valuable nursing resources during tough economic times. | \| *P* \| Nursing workforce in developed countries \| \| --- \| --- \| \| *I* \| Determinants \| \| *C* \| Not stated \| \| *O* \| Turnover \| \| *S* \| Not stated \| | \| 1 \| Developed countries  (by Canada) \| \| --- \| --- \| \| 2 \| 2007-2012 \| \| 3 \| English \| | \| **Total number** \| **23** \| \| --- \| --- \| \| Quantitative \| - \| \| *Experimental (quasi)* \| *-* \| \| *Observational* \| *-* \| \| Qualitative \| - \| \| Mix-Methods \| - \| \| Other \| - \| \| No details available. \| \| | Increased turnover is anticipated with improved economic conditions.  In the short run, economic downturns may temporarily reduce the demand for and increase the supply of nurses, thereby influencing nursing wages and turnover rates. These effects may destabilise the nursing labour market in the long run. (Abstract) | High risk of bias  3/11 |
| Borda  1997 | To identify the main influences on nurses’ turnover and absence and possible common factors influencing both as well as the relationship between absence and turnover. | \| *P* \| Adult nurses \| \| --- \| --- \| \| *I* \| Determinants \| \| *C* \| Not stated \| \| *O* \| Turnover \| \| *S* \| Not stated \| | \| 1 \| International (by Malta) \| \| --- \| --- \| \| 2 \| 1971-1996 \| \| 3 \| English \| | \| **Total number** \| **32** \| \| --- \| --- \| \| Quantitative \| 24 \| \| *Experimental (quasi)* \| *-* \| \| *Observational* \| *24* \| \| Qualitative \| 4 \| \| Mix-Methods \| 4 \| \| Other \| - \| | There is unequivocal support for the relationship between job satisfaction, intent to stay and low turnover. Studies show that intent to stay acts as a mediating variable between the other two variables. The strength of the relationships, however, differs across studies. The model shows job satisfaction to be positively related to intent to stay and inversely related to absence. Intent to stay is inversely related to turnover whilst absence is directly related to turnover. Pay is directly related to intent to stay, whilst employment opportunity is inversely related. Kinship responsibility is directly related to intent to stay and absence for female nurses and inversely related to absence of male nurses. Absence and intent to stay are inversely related. The relationships of pay, kinship responsibility and employment opportunity to intent to stay are weaker than that between job satisfaction and intent to stay, whilst the effect of family responsibilities on absence is stronger than that of job satisfaction on absence. The relationship between intent to stay and turnover is stronger than that between absence and turnover. | High risk of bias  2/11 |
| Daouk-O¨yry  2014 | What are the antecedents and outcomes of absenteeism and turnover among nursing staff in hospital settings? | \| *P* \| Nursing staff in hospital settings \| \| --- \| --- \| \| *I* \| Determinants \| \| *C* \| Not stated \| \| *O* \| Turnover \| \| *S* \| Peer-reviewed primary studies \| | \| 1 \| International (by Lebanon) \| \| --- \| --- \| \| 2 \| 2007 – 2013 \| \| 3 \| English \| | \| **Total number** \| **41** \| \| --- \| --- \| \| Quantitative \| 33 \| \| *Experimental (quasi)* \| *-* \| \| *Observational* \| *33* \| \| Qualitative \| 4 \| \| Mix-Methods \| 4 \| \| Other \| - \| | Consistent with the literature reviewed in this paper, staff nurse turnover and absenteeism is influenced by macro (i.e., job, organizational, and national) as well as micro (i.e., individual and interpersonal) level variables.  The causes of absenteeism and turnover as individual behaviours can be better perceived as resulting from properties of the whole healthcare system within which nurses operate. Comprehensively understanding such individual behaviours may therefore require analysis of individual characteristics, as well as, those of the social group(s) they belong to (Diez-Roux, 1998). Multilevel research has the potential of facilitating this perspective, and allowing researchers and practitioners to conceive turnover and absenteeism as resulting from relational properties rather than mere aggregates of individuals.  We attempted to integrate all the available evidence into a systematic conceptual JOINT model (Job, Organization, Individual, National and interpersonal). From an interventional perspective, the proposed conceptual JOINT model can provide nurse managers with a new lens for investigating and managing attendance behaviour in their organizations. From a theoretical perspective, the JOINT model was designed from a multilevel lens in order to serve as guiding framework for researchers interested in capturing absenteeism and turnover from a multi-dimensional perspective. | High risk of bias  4/11 |
| Duffield 2014 | To compare turnover rates and costs from four studies in four countries (US, Canada, Australia, New Zealand) that employed the original NTCCM (Nursing Turnover Cost Calculation Methodology). A secondary aim is to compare the relative weightings of individual cost items to determine whether some components of this costing methodology have changed over time or across countries. | \| *P* \| Hospital units \| \| --- \| --- \| \| *I* \| Consequences \| \| *C* \| Not stated \| \| *O* \| Turnover costs (societal, system) \| \| *S* \| Papers that reported on both costs and rates of nurse turnover and to those that used the original NTCCM \| | \| 1 \| Developed Countries  (by Australia) \| \| --- \| --- \| \| 2 \| No limits \| \| 3 \| English \| | \| **Total number** \| No details available. \| \| --- \| --- \| \| Quantitative \| \| *Experimental (quasi)* \| \| *Observational* \| \| Qualitative \| \| Mix-Methods \| \| Other \| | There is limited data relating to what attracted this generation to nursing or what might retain them in the nursing workforce.  Developing an understanding of what attracts the Y Gen to nursing, what managers can do to retain the Y Gen in nursing and how the nursing profession can support the Y Gen to assume a role in nursing and nursing governance will ensure that the retiring generation has left the nursing profession in capable hands. | High risk of bias  3/11 |
| Duvall 2010 | What factors are contributing to this nursing shortage? [recognising current demand-supply imbalances and looming retirement of a significant portion of the workforce] | \| *P* \| Nurses in the United States \| \| --- \| --- \| \| *I* \| Determinants & consequences \| \| *C* \| Not stated \| \| *O* \| Retention/Intention to leave  Turnover,  Impact of shortages on patient care \| \| *S* \| Not stated \| | \| 1 \| USA \| \| --- \| --- \| \| 2 \| 2000-2007 \| \| 3 \| English \| | \| **Total number** \| **27** \| \| --- \| --- \| \| Quantitative \| 22 \| \| *Experimental (quasi)* \| *-* \| \| *Observational* \| *22* \| \| Qualitative \| 3 \| \| Mix-Methods \| 2 \| \| Other \| - \| | The stressors associated with the practice of nursing appear to affect nurses both physically and psychologically. In addition, increasing workloads have the potential to negatively affect both the satisfaction of staff and the quality of patient care. These factors are contributing to the exodus of nurses from the profession. To attract and keep new nurses, efforts must be made to improve the perception of nursing as a career and effectively mentor their transition from the academic environment. | High risk of bias  4/11 |
| Ellenbecker 2011 | To examine/test a theoretical relationship of organizational commitment (affective, continuance) and home healthcare nurse retention | \| *P* \| Home healthcare nurses \| \| --- \| --- \| \| *I* \| Determinants & consequences \| \| *C* \| Not stated \| \| *O* \| Nurse retention and intent to stay;  Impact on quality of patient care \| \| *S* \| Articles reporting original research, concepts or theories pertaining to the research aim \| | \| 1 \| International (by USA) \| \| --- \| --- \| \| 2 \| 1990- May 2011 \| \| 3 \| English \| | \| **Total number** \| **26** \| \| --- \| --- \| \| Quantitative \| 22 \| \| *Experimental (quasi)* \| *-* \| \| *Observational* \| *22* \| \| Qualitative \| 1 \| \| Mix-Methods \| 2 \| \| Other \| - \| | Understanding retention based on organizational commitment suggests that strategies to increase nurses’ affective commitment to the organization through interventions to decrease job stress and improve job satisfaction will most likely have a positive effect on nurse work behaviours and ultimately on patient care.  The proposed model hypothesizes the relationship of nurse organizational commitment, specifically continuance commitment, and individual nurse characteristics of living arrangement and tenure. | Moderate risk of bias  6/11 |
| Hayes  2006 | To examine the current state of knowledge about the scope of the nurse turnover problem, definitions of turnover, factors considered to be determinants of nurse turnover, turnover costs and of most importance to the authors, the impact of turnover on patient, nurse and system outcomes. | \| *P* \| Registered or practical/enrolled nurses working in the hospital, long-term or community care areas \| \| --- \| --- \| \| *I* \| Determinants & consequences \| \| *C* \| Not stated \| \| *O* \| Turnover, intention to quit / Turnover intention / anticipated turnover, Intent/commitment to remain,  Retention \| \| *S* \| Peer-reviewed primary studies \| | \| 1 \| International (by Canada) \| \| --- \| --- \| \| 2 \| Late 1990s- onwards \| \| 3 \| English \| | \| **Total number** \| **37** \| \| --- \| --- \| \| Quantitative \| 34 \| \| *Experimental (quasi)* \| *-* \| \| *Observational* \| *34* \| \| Qualitative \| 3 \| \| Mix-Methods \| - \| \| Other \| - \| | DETERMINANTS.  The factors associated with nurse turnover, job dissatisfaction and expressed intent to leave are most consistently reported as impacting turnover… to be influenced by certain moderators including professional commitment and personal disposition. Organizational characteristics associated with workload, management style, empowerment and autonomy, promotional opportunities and work schedules are believed to contribute to turnover, and therefore researchers suggest that administrative interventions to improve quality of work life are imperative for long-term resolution. While socio-demographic characteristics are not usually considered explanatory in turnover behavior, and related findings have been somewhat inconsistent, factors such as younger age, inexperience and fewer years of the job, higher educational level, and kinship responsibilities are claimed to predispose nurses to turnover. In terms of economic factors, findings relating to the impact of remuneration on turnover are varied (244).  CONSEQUENCES. Financial costs of replacing individual nurses are being estimated, however, studies have yet to produce evidence in terms of incremental staffing costs and health care system costs. Studies that examine the effect of inadequate nurse staffing and poor work environments on nurse health and safety outcomes often do not include variables explicit to nurse turnover. Similarly, few studies have produced findings that link nurse turnover to measurable patient outcomes (246). | High risk of bias  4/11 |
| Hayes  2012 | As a follow-up to a previous literature review to examine the issue of nursing turnover, its determinants and impact on patient, nurse and system outcomes (Hayes et al., 2006), this paper summarizes subsequent research and describes ongoing methodological challenges and implications of new evidence for future studies. | \| *P* \| Registered or practical/enrolled or assistant nurses working in the hospital, long-term or community care areas. \| \| --- \| --- \| \| *I* \| Determinants & consequences \| \| *C* \| Not stated \| \| *O* \| Turnover  Turnover intent \| \| *S* \| Not stated \| | \| 1 \| International (by Canada) \| \| --- \| --- \| \| 2 \| 2009 onwards \| \| 3 \| English \| | \| **Total number** \| **60** \| \| --- \| --- \| \| Quantitative \| 60 \| \| *Experimental (quasi)* \| *-* \| \| *Observational* \| *60* \| \| Qualitative \| - \| \| Mix-Methods \| - \| \| Other \| - \| | A comprehensive search of recently published studies about nursing turnover was conducted as a follow-up to a previous literature review to examine its determinants and impact on patient, nurse and system outcomes. Research continues to be hindered by methodological challenges, and studies mostly focus on determinants of nurse turnover with evidence lacking as to the impact of actual turnover behaviour. Recent studies offer insight into generational factors that should be considered in strategies to promote stable staffing in healthcare organizations. Nursing turnover continues to present serious challenges at all levels of health care.  Longitudinal research is needed to produce new evidence of the relationships between nurse turnover and related costs, and the impact on patients and the health care team. | High risk of bias  3/11 |
| Hutchinson  2012 | This paper explores the literature related to attracting the Y Generation (Y Gen) to the nursing profession and retaining them in our current workforce. | \| *P* \| Y generation (1980-2000) actual or potential nursing workforce \| \| --- \| --- \| \| *I* \| Determinants \| \| *C* \| Not stated \| \| *O* \| Recruitment, retention and turnover of Y Gen nurse \| \| *S* \| Not stated \| | \| 1 \| International (by Australia) \| \| --- \| --- \| \| 2 \| Not specified \| \| 3 \| English \| | \| **Total number** \| No details available. \| \| --- \| --- \| \| Quantitative \| \| *Experimental (quasi)* \| \| *Observational* \| \| Qualitative \| \| Mix-Methods \| \| Other \| | There is limited data relating to what attracted this generation to nursing or what might retain them in the nursing workforce. Developing an understanding of what attracts the Y Gen to nursing, what managers can do to retain the Y Gen in nursing and how the nursing profession can support the Y Gen to assume a role in nursing and nursing governance will ensure that the retiring generation has left the nursing profession in capable hands. | High risk of bias  3/11 |
| Lim  2010 | To explore the nursing literature on Singaporean nurses’ experiences of stress and the strategies they use to cope with this stress in the context of the Singaporean health-care system.  Specifically, it aims to identify: (i) stress levels and types of stressors experienced; (ii) effects of stressors; and (iii) the various coping strategies used. | \| *P* \| Nurses in Singapore \| \| --- \| --- \| \| *I* \| Determinants \| \| *C* \| Not stated \| \| *O* \| Intention to leave \| \| *S* \| Any primary research paper \| | \| 1 \| Singapore \| \| --- \| --- \| \| 2 \| 1997 - 2007 \| \| 3 \| English \| | \| **Total number** \| **9** \| \| --- \| --- \| \| Quantitative \| 9 \| \| *Experimental (quasi)* \| *-* \| \| *Observational* \| *9* \| \| Qualitative \| - \| \| Mix-Methods \| - \| \| Other \| - \| | The published studies paint a picture of Singaporean nurses’ experiences related to workplace stress and interpersonal relationships, particularly with patients and colleagues. These stressors can lead to poor health and daily functioning. Nurses also experience job dissatisfaction and report their intention to change profession as a result of work-related stress. The literature also indicates that Singaporean health organizations can work on resolving these issues and increase effectiveness of coping strategies used by nurses. | High risk of bias  4/11 |
| Storey  2012 | To examine the reasons of why older nurses choose to leave primary or community care and the factors that might encourage them to stay. | \| *P* \| Older nurses in primary and community care \| \| --- \| --- \| \| *I* \| Determinants \| \| *C* \| Not stated \| \| *O* \| Factors influencing nurse retention and intention to leave \| \| *S* \| Not stated \| | \| 1 \| International \| \| --- \| --- \| \| 2 \| 1995– 2006 \| \| 3 \| English \| | \| **Total number** \| No details available. \| \| --- \| --- \| \| Quantitative \| \| *Experimental (quasi)* \| \| *Observational* \| \| Qualitative \| \| Mix-Methods \| \| Other \| | With high numbers of nurses over the age of 50 in primary and community care services, it is vital to understand the factors that influence retention. The barriers to the retention of older nurses were summed up by Meadows (2002) as unwillingness to confront the managerial complexities of implementing older workers initiatives, lack of investment in human resources, and inflexibility. Discrimination, poor health, physically and technologically demanding work environments and financial considerations can also be demonstrated (Moseley et al. 2008). | High risk of bias  3/11 |
| Tai  1998 | To present: (1) a review of nursing turnover studies over the twenty year period from 1977 to 1996; and (2) a summary of associations of turnover behavior with nursing employees' socio-demographic factors, facility organizational characteristics, and social support characteristics | \| *P* \| Registered nurses, licensed vocational nurse, and licensed practical nurse) \| \| --- \| --- \| \| *I* \| Determinants & consequences \| \| *C* \| Not stated \| \| *O* \| Turnover  costs \| \| *S* \| Not stated \| | \| 1 \| International (by USA) \| \| --- \| --- \| \| 2 \| 1977 - 1996 \| \| 3 \| English \| | \| **Total number** \| **37** \| \| --- \| --- \| \| Quantitative \| 37 \| \| *Experimental (quasi)* \| *-* \| \| *Observational* \| *37* \| \| Qualitative \| - \| \| Mix-Methods \| - \| \| Other \| - \| | The conflicting results obtained from simple bivariate analyses underscored the complexity of turnover behavior. Rigorously controlled investigations are now being used to better understand a health care employee's decision to leave his or her job. From the many research studies investigating why nurses leave their jobs, some variables are beginning to emerge as significant predictors of turnover behavior. Studies on turnover in hospitals and nursing homes have shown that older staff, minorities, those with higher income, or longer tenure were associated with lower actual or intended turnover. Job satisfaction and tension, organizational commitment, perceived job possibilities, and supervisor's behavior were other factors that may lead to turnover. | High risk of bias  3/11 |

PICOS: Population, Intervention, Comparison, Outcomes, Study design; P: Population; I: Intervention; C: Comparison; O: Outcome; S: Study design
